# BMI Not WHR Modulates BOLD fMRI Responses in a Sub-Cortical Reward Network When Participants Judge the Attractiveness of Human Female Bodies

**DOI:** 10.1371/journal.pone.0027255

**Published:** 2011-11-15

**Authors:** Ian E. Holliday, Olivia A. Longe, N. Jade Thai, Peter J. B. Hancock, Martin J. Tovée

**Affiliations:** 1 Aston Brain Centre, Aston University, Birmingham, England, United Kingdom; 2 Psychology Department, University of Stirling, Stirling, Scotland, United Kingdom; 3 Institute of Neuroscience, Newcastle University, Newcastle, England, United Kingdom; University Medical Center Groningen UMCG, The Netherlands

## Abstract

In perceptual terms, the human body is a complex 3d shape which has to be interpreted by the observer to judge its attractiveness. Both body mass and shape have been suggested as strong predictors of female attractiveness. Normally body mass and shape co-vary, and it is difficult to differentiate their separate effects. A recent study suggested that altering body mass does not modulate activity in the reward mechanisms of the brain, but shape does. However, using computer generated female body-shaped greyscale images, based on a Principal Component Analysis of female bodies, we were able to construct images which covary with real female body mass (indexed with BMI) and not with body shape (indexed with WHR), and vice versa. Twelve observers (6 male and 6 female) rated these images for attractiveness during an fMRI study. The attractiveness ratings were correlated with changes in BMI and not WHR. Our primary fMRI results demonstrated that in addition to activation in higher visual areas (such as the extrastriate body area), changing BMI also modulated activity in the caudate nucleus, and other parts of the brain reward system. This shows that BMI, not WHR, modulates reward mechanisms in the brain and we infer that this may have important implications for judgements of ideal body size in eating disordered individuals.

## Introduction

In perceptual terms, the human body is an evolutionarily important, complex 3D shape. It potentially conveys a wide range of information, including information important for human mate selection. Behavioural studies have shown that both male and female observers are consistent and reliable in their ratings of the attractiveness of female bodies [1], [2]. It is likely that the perception of attractive bodies is linked to the reward systems in the brain. Several studies have shown that processing pleasant pictures differs from neutral pictures by activation in regions including the anterior cingulate, left precuneus, right and left insula, right inferior frontal gyrus, and left caudate nucleus [3]–[5]. Additionally, when observers judge facial attractiveness functional imaging shows an activation of the medial orbitofrontal cortex, left anterior frontal cortex, left frontal-temporal junction, nucleus accumbens, right caudate nucleus, and visual cortex [6]–[8]. This pattern of activation is likely to reflect the positive reward properties of faces. The role of the orbitofrontal cortex and the striatum in processing reward-based stimuli has been extensively documented [6], [9] and it is likely that an observer's preference for an attractive face is mediated by its reward value. We therefore hypothesized the attractiveness preferences for bodies would activate the same reward systems.

The two features of the body most frequently used to explain attractiveness judgments are overall body fat, indexed by the body mass index (BMI) (e.g. [1], [10], [11]) and the specific distribution of fat deposition on the lower body, indexed by the ratio of waist circumference to hip circumference — the waist-to-hip ratio (WHR) (e.g. [12], [13]. The role of BMI in attractiveness judgements is particularly important, as it is an over-estimation of body mass that systematically shifts the ideal body size of eating disordered women towards a lower body weight (i.e. they see themselves as bigger than they are, which produces a dissatisfaction with their body size), which in turn drives their restrictive dietary behaviour (e.g. [14]–[16]).

Under normal circumstances, BMI and WHR co-vary in Caucasian populations [17]. For example, the Health Survey for England [18], which includes directly obtained measurements from 1808 Caucasian women of reproductive age (16–45) ranging in BMI from around 15–45, shows a correlation between BMI and WHR of 0.45. That BMI and WHR and other related physical variables are correlated is not surprising. As a body adds fat, the circumference of the waist and hips also increases; BMI is strongly correlated with both waist circumference (Pearson Correlation, r = 0.87, p<0.0001) and the hip circumference (r = 0.90, p<0.0001). WHR is also correlated with weight (r = 0.40, p<0.0001) and to a lesser extent height (r = 0.12, p<0.0001) [18]. For studies of attractiveness, the correlation between features such as BMI and WHR raises the problem of collinearity amongst explanatory variables, and raises the question of whether WHR or BMI is the primary cue used in attractiveness judgements.

In an attempt to avoid this problem, a recent fMRI study used before and after photographs of the lower torsos (from the bottom of the ribcage to half way down the thigh) of 7 women who had undergone a cosmetic surgical procedure [19]. The surgery involved moving adipose tissue from the stomach to the hips and thighs, and it is reported that this changed WHR but made no statistically significant change to BMI. These images appeared to activate regions which are associated with neural reward mechanisms (such as the anterior cingulate cortex and nucleus accumbens). It was suggested that the changing WHR of these images modulated the activity in the reward systems, but by contrast, changing BMI had no effect. However, this study had two serious flaws. Firstly, there were potential problems with the images. The photographs were not standardised and vary in viewing angle (varying between a profile view and a view-point behind the body) and illumination in the before and after conditions, so although the women in these pictures are suggested not to vary significantly in BMI in the before and after surgery pictures, they may *appear* to alter in their BMI. Moreover, both behavioural and eye-movement studies suggest that the degree of stomach depth (i.e. the degree to which the stomach protrudes) is used as a key cue to judge BMI [10], [20], [21]. This surgical intervention, which artificially alters this part of the body, may lead observers to perceive a difference in BMI in the before and after condition. This is important because, the observers have only the visual image to go on, and if the image appears to vary in BMI (even if there is no significant change in the BMI of participants in the photographs) then the observers will react to the images as though they do alter in BMI. Thus, the *apparent* BMI and WHR of the pictures may co-vary and it is not clear whether the reported changes in neural activity were due to changes in WHR, apparent BMI or some mixture of the two. Secondly, it appears that no correction factor was applied to the BOLD activity reported in their paper, to compensate for multiple statistical testing in the analysis. Such an analysis is not without precedent. A number of published studies have also not used corrections factors, but from a purely statistical standpoint, without such correction factors BOLD activations cannot be said to be statistically significant [22].

It is possible to construct a set of artificial female bodies using four independent descriptors of shape derived from a principal components analysis (PCA) of real body shape [1]. The body mass of these bodies can be quantified using the perimeter-to-area ratio (PAR) [21], [23]. The PCA allows a separation of the different components of body size and shape, and allows the construction of sets of bodies which vary in BMI but not in WHR, and in WHR but not BMI. We combined the behavioural rating of these images with an fMRI study to determine whether changing BMI or WHR does modulate activity in the reward areas of the brain and to establish a foundation for studies on patients with disordered perception of body size and shape, such as patients with Anorexia and Bulimia Nervosa.

## Methods

### Ethics Statement

Ethical approval was given for this study from the Aston University Human Sciences Ethical Committee and was conducted in accordance with the guidance given in The Declaration of Helsinki [24]. Participants received information about the study including its purpose, explaining what their participation would involve and explaining their right not to participate and to withdraw from the study at any time. Each participant gave their written consent to participation before the study they took part in commenced.

### Participants

12 Participants were recruited from the student and staff population at Aston University (6 were male and 6 were female). Each participant was screened for their safety and health according to a protocol approved by Aston University Human Sciences Ethical Committee 24 Hrs before attending, and again immediately prior to, their scanning session. Before the fMRI scan, participants were shown the stimulus sequence running and used the button response to practice rating one or two images on a computer screen outside the scanner room. They were instructed to provide their initial reaction and reassured that the images were computer generated images and not photographs of real women who could potentially be upset by the ratings.

### Behavioural Methods

**Figure 1:**
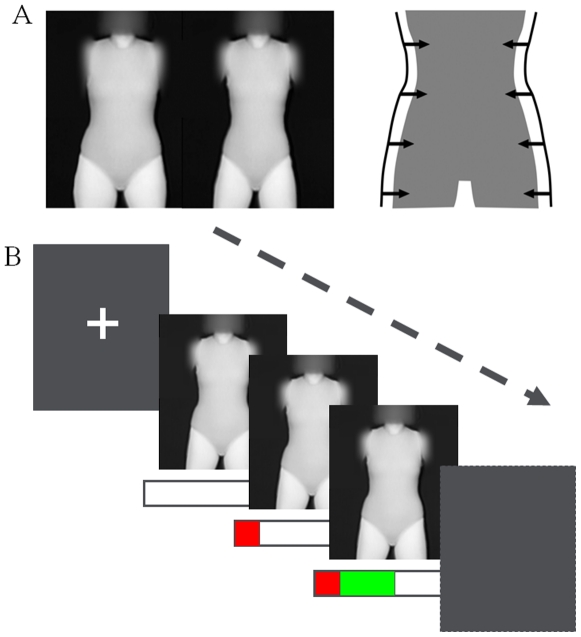
Female Body Shape Stimuli and experimental design. A): Sample images ranged from a low value of PAR (left) to a high value (right). There were 40 such images with different PAR values spanning a range corresponding to the full range of body shapes typical of the normal BMI range for young female adults. B): The stimulus sequence: Fixation cross (1, 2 or 3s); Body image appears with empty rating bar; after 2s of body attractiveness assessment the rating bar started to fill in red alerting participant to the approaching reporting phase; 200ms later the fill colour changed to green after which a rating of attractiveness could be made by pressing a response button, short green segments signalling low attractiveness ratings, and long green segments signalling high attractiveness ratings, the green bar filled completely in 2s; 7s after first appearing the stimulus offset to a blank screen followed by a random delay before the next trial started, indicated by fixation re-appearance. Total trial durations were, 16, 18 or 20 s.

During the experiment participants viewed grey scale images of artificial female figures presented via a back projector onto a screen mounted at the end of the MR scanner. The stimuli (N = 40) were generated from a principal components analysis of images of real figures [1], and an accurate estimate of the BMI of the bodies can be calculated using PAR [23]. This estimate is referred to as BMI_PAR_. In this study, BMI_PAR_ varied from 20.9 to 23.3 (limits that are well within the normal BMI range of 18.5–24.9). We varied the WHR range from 0.65 to 0.77. Examples of two figures and the effect of varying PAR are shown in figure 1A.

### Procedure

Refer to figure 1B showing the stimulus sequence. In an event-related design, the body images were displayed for 7 seconds, appearing after a fixation cross of variable duration (1–3 seconds). The rating phase started 2 seconds after the appearance of the stimulus body image with the appearance of an animated scale bar at the bottom of the screen. The scale bar started filling with red from the left hand end of the bar to alert the participants to prepare to deliver their attractiveness rating. Then 200 ms later, the scale bar started to fill with green, indicating that a response could be made. Participants pressed the response button when the proportion of green filling matched their attractiveness rating, and the bar stopped filling. If the bar was allowed to completely fill with green it took about 2 seconds, representing the most attractive response rating. At the end of the 7 second stimulus display time, the screen was blanked to black. The blank inter-stimulus interval (ISI) continued until the fixation cross appeared again to cue the next trial. The length of the ISI varied between 6 and 12 s.

### FMRI Methods

All MRI images were collected using a 3-Tesla Siemens Magentom Trio MRI Scanner at Aston University. T2* weighted gradient echo sequences were acquired with the following parameters: TR = 3000ms, TE = 60ms, 64x64 matrix of 3x3mm in plane resolution, 44 slices of 3mm thickness per whole brain volume. 241 volumes were collected in a total experiment run-time of about 12 minutes. In addition, an anatomical volume was acquired using MP-RAGE inversion recovery sequence with GRAPPA, 256 x256 matrix, 1x1x1 mm voxels and 176 slices.

### Analysis

FMRI images were analyzed using SPM2 (The Wellcome Trust Centre for Neuroimaging http://www.fil.ion.ucl.ac.uk/spm/). The images were realigned, normalised to the MNI template brain and smoothed to 8mm FWHM. The following events of interest were modelled with the canonical haemodynamic response function (HRF) for each participant for the first level analysis: fixation onset, body image onset (BODY), and the scale bar onset (JUDGE). Covariates of interest included in the model design were the participant rating (RATING), the body image BMI index (BMI_par_) and the body image WHR index (WHR). The main focus of the analyses were the separate regressions of the BOLD activation associated with the BODY event and the body image shape (BMI_PAR_), body image waist-to-hip ratio (WHR), and participant attractiveness ratings (RATING). The statistical contrast images (T statistic images) for each participant were then entered into the second level group analysis. For the fMRI results the statistical threshold for detection of a significant response across the group of participants was set at a false discovery rate (FDR) of (p<0.05) corrected for the whole brain volume, and additionally the minimum size of cluster(k) accepted was typically K > = 8 for an uncorrected cluster significance cPu<0.05.

## Results

### Behavioural Results

**Figure 2:**
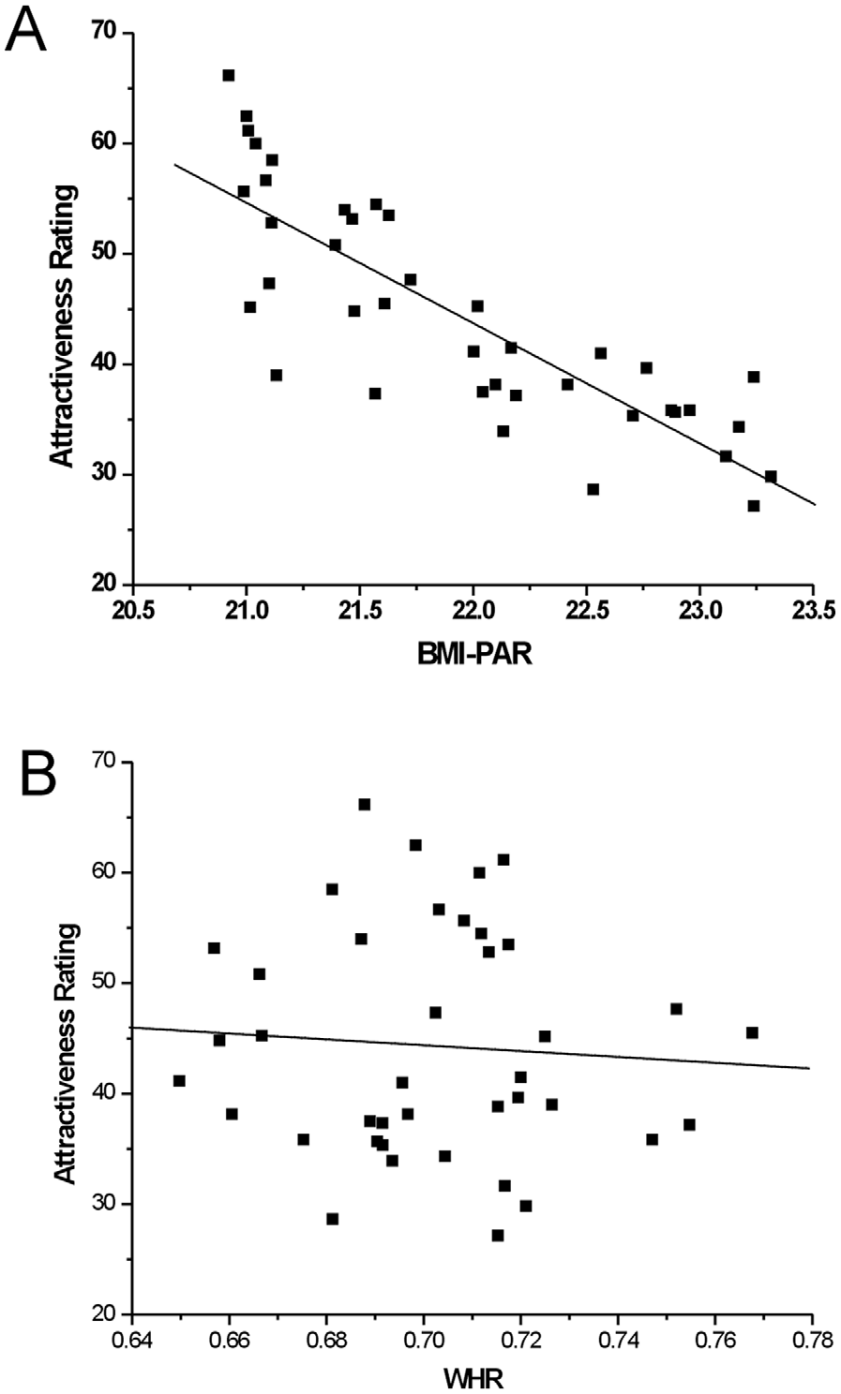
Participant attractiveness ratings: correlation with BMI and WHR. A)Plot of average attractiveness ratings against the BMI_PAR_ of the bodies in the photographs. B)Plot of average attractiveness ratings against the WHR of the bodies in the photographs.

The behavioural results (Figure 2) show a negative correlation of attractiveness ratings with BMI_PAR_ (r = −0.84, P<0.0001). However, there was no significant correlation of attractiveness with WHR (r = −0.07, p = 0.666).

### Negative Regression of BMI_PAR_ with Body Event

**Figure 3:**
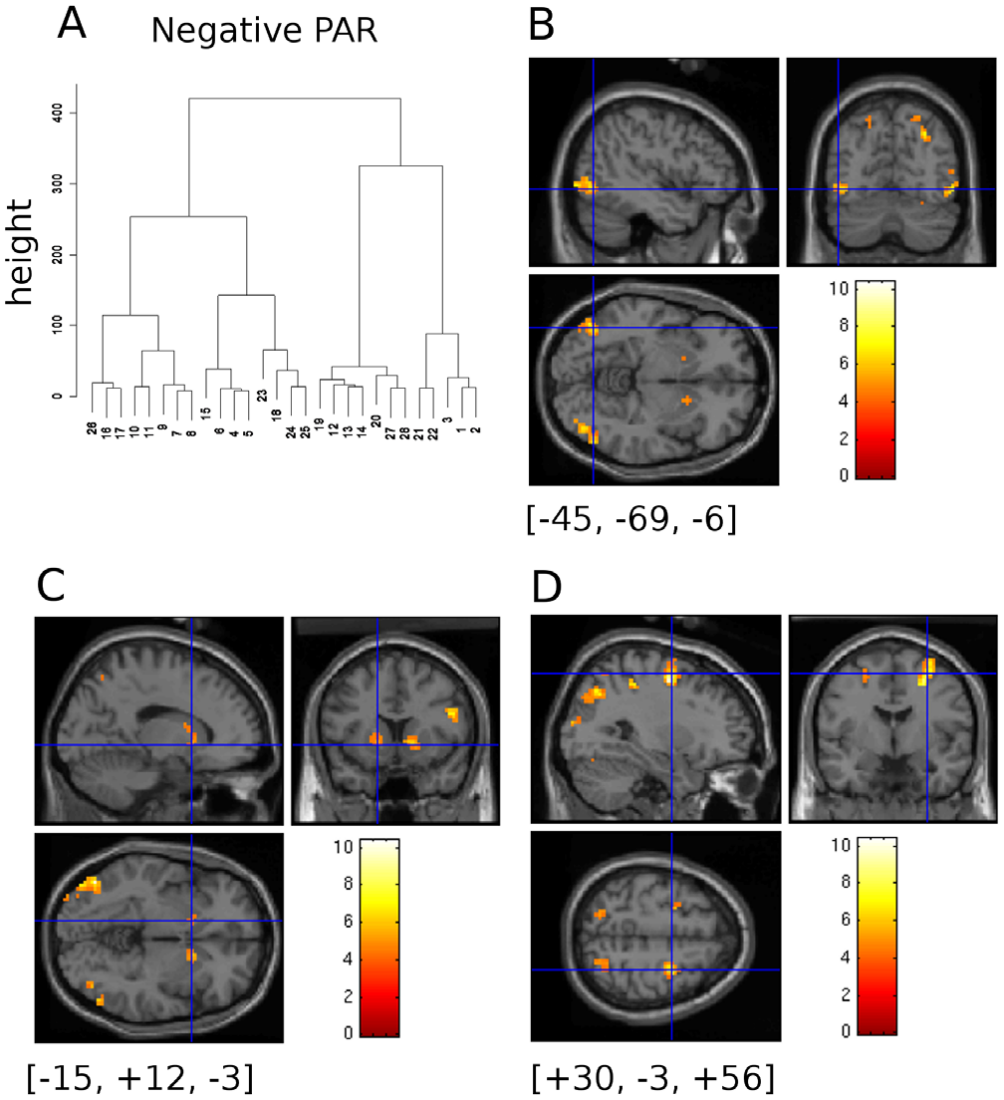
Negative Regression of BMI_PAR_ with BOLD. A)Cluster dendrogram for (X,Y,Z) peak BOLD activations sites for the negative regression of BMI_PAR_ with BOLD signal intensity based on a linear distance measure computed on the (Talairach transformed) co-ordinates of significant peaks of BOLD activation. Dendrograms in the following figures were computed this way too. B)Negative regression of BMI_PAR_ on BOLD intensity. Activations are superimposed on the Colin27 template supplied with SPM2 courtesy of the Montreal Neurological Institute. Cross hairs located at [−45, −69, −6] (untransformed MNI co-ordinates) show the putative location of the EBA in the left hemisphere; there is a corresponding activation in the right hemisphere. C)Bilateral caudate activation is visible at the cross hair position [−15, +12, −3] (untransformed MNI co-ordinates) and the corresponding position in the right hemisphere. D)A superiorly positioned slice selection shows frontal eye fields and bilateral posterior parietal activity. Crosshair location [+30, −3 ,+56] untransformed coordinates.

Table 1 shows the results of a negative regression of the BOLD signal associated with the BODY event and the BMI_PAR_ values. The locations of activations reported in the table (columns X, Y, Z in mm) were transformed from those reported from the SPM2 software using the procedure of [25] to obtain more accurate reference to Talairach and Tourneaux atlas co-ordinates [26]. Anatomical identification was obtained by submitting the transformed co-ordinates to the Talairach Daemon Java client [27]. The X,Y & Z co-ordinates were additionally clustered using hierarchical k-means clustering in R using a minimum variance distance measure (procedure hlust(), http://www.r-project.org/), and the resulting tree cut to yield 9 clusters, shown in table 1 in column Cl and labelled 1 to 9 . This gives broadly the same clustering as provided by SPM2, but provides some additional structuring of the activations. Within the clusters the ordering of the activations was retained as in the original SPM output. Column Sx in table 1 provides an ordered index to allow readers to re-sort the table into the original ordering from SPM2 if required (Figure 3a).

**Table 1:** Negative regression of BODY and BMI_PAR_.

Cl	Sx	K	cPu	FDR	T	EqZ	X	Y	Z	H	Structure	BA
1	1	194	0	0.007	10.39	5.03	−*45.58*	−*68.35*	−*5.67*	L	Middle Occipital Gyrus	BA 37
1	2	(194)		0.007	9.6	4.87	−48.51	−74.97	4.46	L	Middle Occipital Gyrus	BA 19
1	3	(194)		0.014	7.72	4.44	−31.92	−86.5	6.35	L	Middle Occipital Gyrus	BA 18
2	4	59	0	0.007	10.25	5	43.1	4.04	29.71	R	Inferior Frontal Gyrus	BA 9
2	5	(59)		0.021	6.24	4	40.24	−2.06	34.49	R	Precentral Gyrus	BA 6
2	6	(59)		0.022	5.91	3.89	31.95	1.04	31.94	R	Precentral Gyrus	BA 6
2	15	30	0	0.014	7.01	4.24	40.46	29.73	26.7	R	Middle Frontal Gyrus	BA 9
3	7	83	0	0.007	9.83	4.92	28.86	−39.64	44.25	NG		
3	8	(83)		0.014	7.65	4.42	31.7	−44.72	38.41	R	Inferior Parietal Lobule	BA 40
3	9	(83)		0.026	5.69	3.81	42.75	−36.92	44.74	R	Inferior Parietal Lobule	BA 40
4	10	89	0	0.007	9.56	4.86	26.13	−11.94	49.53	R	Precentral Gyrus	BA 6
4	11	(89)		0.016	6.65	4.13	28.73	−10.47	63.23	R	Precentral Gyrus	BA 6
5	12	91	0	0.013	7.79	4.45	40.68	−59.11	−16.85	RCb	Declive	*
5	13	(91)		0.014	6.91	4.21	*48.86*	−*65.78*	−*6.53*	R	Middle Occipital Gyrus	BA 37
5	14	(91)		0.017	6.52	4.09	37.73	−74.11	−7.51	R	Inferior Occipital Gyrus	BA 19
5	19	8	0.032	0.024	5.81	3.85	24	−67.4	−17.92	RCb	Declive	*
5	20	14	0.007	0.024	5.78	3.84	26.35	−87.06	9.99	R	Middle Occipital Gyrus	BA 19
5	27	9	0.025	0.041	4.68	3.4	48.68	−67.09	6.85	R	Middle Temporal Gyrus	BA 37
5	28	(9)		0.043	4.58	3.36	37.57	−69.83	6.41	R	Middle Occipital Gyrus	BA 19
6	16	43	0	0.014	6.98	4.23	26.12	−66.79	33.52	R	Precuneus	BA 7
6	17	(43)		0.038	4.93	3.51	26.13	−77.71	29.79	R	Cuneus	BA 7
6	26	24	0.001	0.032	5.25	3.64	20.39	−68.07	46.82	R	Precuneus	BA 7
7	18	33	0	0.021	6.14	3.97	12.92	9.6	2.7	R	Caudate	Caudate Head
7	24	36	0	0.031	5.33	3.67	−14.87	9.48	4.92	L	Lentiform Nucleus	Putamen
7	25	(36)		0.033	5.19	3.62	−17.83	5.4	18	L	Caudate	Caudate Body
8	21	27	0	0.025	5.71	3.82	−24.02	−65.05	46.35	L	Superior Parietal Lobule	BA 7
8	22	(27)		0.043	4.6	3.37	−21.11	−66.81	35.43	L	Precuneus	BA 7
9	23	12	0.011	0.026	5.67	3.8	−23.9	−6.62	54.59	L	Sub-Gyral	BA 6

The locations of activations reported in the table (columns X, Y, Z in mm) were transformed using the procedure of Lancaster et al. 2007 from those reported from the SPM2 software to obtain more accurate reference to Talairach and Tourneaux atlas co-ordinates (Talairach and Tounoux, 1988). The columns left to right are: Cl - the cluster index produced by the hclust algorithm; Sx - the sequence index produced by the SPM2 analysis and for reference to the dendrogram of figure 3; K - the cluster extent associated with activations, in voxels; cPu - the uncorrected cluster level type 1 error rate; FDR –false discovery rate corrected for multiple comparisons; T- t-values of the activations; EqZ - equivalent Z scores; X, Y, Z; H – Hemisphere or subcortical region; Structure –brain anatomical label reported by the Talairach Deamon java client [27] (NG  =  No Grey; RCb – right cerebellum); BA – Brodmann area (*  =  not in the cortex). The two sets of coordinates underlined and italicised are the Left and Right hemispheric activations that we identified in this study that were similarly located to other reports of an extra-striate body area.

NB 9 clusters vs. 8 were chosen as this divides the precentral cluster from IPL.

Cluster 7 of table 1 shows regions of the caudate and lenticulate nucleus bilaterally where BOLD signal increased with decreasing BMI_PAR_. Lower BMI_PAR_ values indicate slimmer body shapes and these were rated as more attractive by our participants, so increasing attractiveness, indexed by BMIpar, increases activity within the caudate and lenticulate nuclei.

There was also extensive activation of visual cortical regions within the occipital lobe, but of chief interest to us are the two regions underlined and italicised in table 1: clusters 1(Sx = 1) and 5(Sx = 13). These were the largest clusters of activation observed; they were located in the middle occipital gyrus. The crosshairs in figure 3B are centred at the largest activation (−45.6, −68.4, −5.7), identified in table 1 as cluster 1(Sx = 1); we believe this is the left hemispheric location of the extrastriate body area (EBA), BA37 (cf.[28], [29]). Its companion activation visible in the right hemisphere (cluster 5(Sx = 12)) was located just below the cortical surface but the activated region extends into the cortex and the second cluster sub-peak 5(Sx = 13) lies in the region of the EBA in the right occipital lobe (48.9, −65.8, −6.5), BA 37. The balance of the other brain activity revealed with this analysis reflects extensive visual activations in the occipital lobe – Clusters 1 & 5, BA 18 & 19 – and the occipito-parietal cortex – clusters 3, 6, & 8 BA40 & BA7. Frontal regions, possibly the frontal eye fields, are also engaged in the task (Figure 3D, axial slice, and cluster 2, 4 & 9, BA6- also compare this image with figure 6 from[30]) all of which may reflect the relatively high level visual judgement and visuo-motor planning needed to perform the task. The results show that brain regions responsive to body-shape and size can be modulated by artificially produced body-shaped stimuli with varying BMI_PAR_.

### Positive Regression of BMI_PAR_ with Body

**Figure 4:**
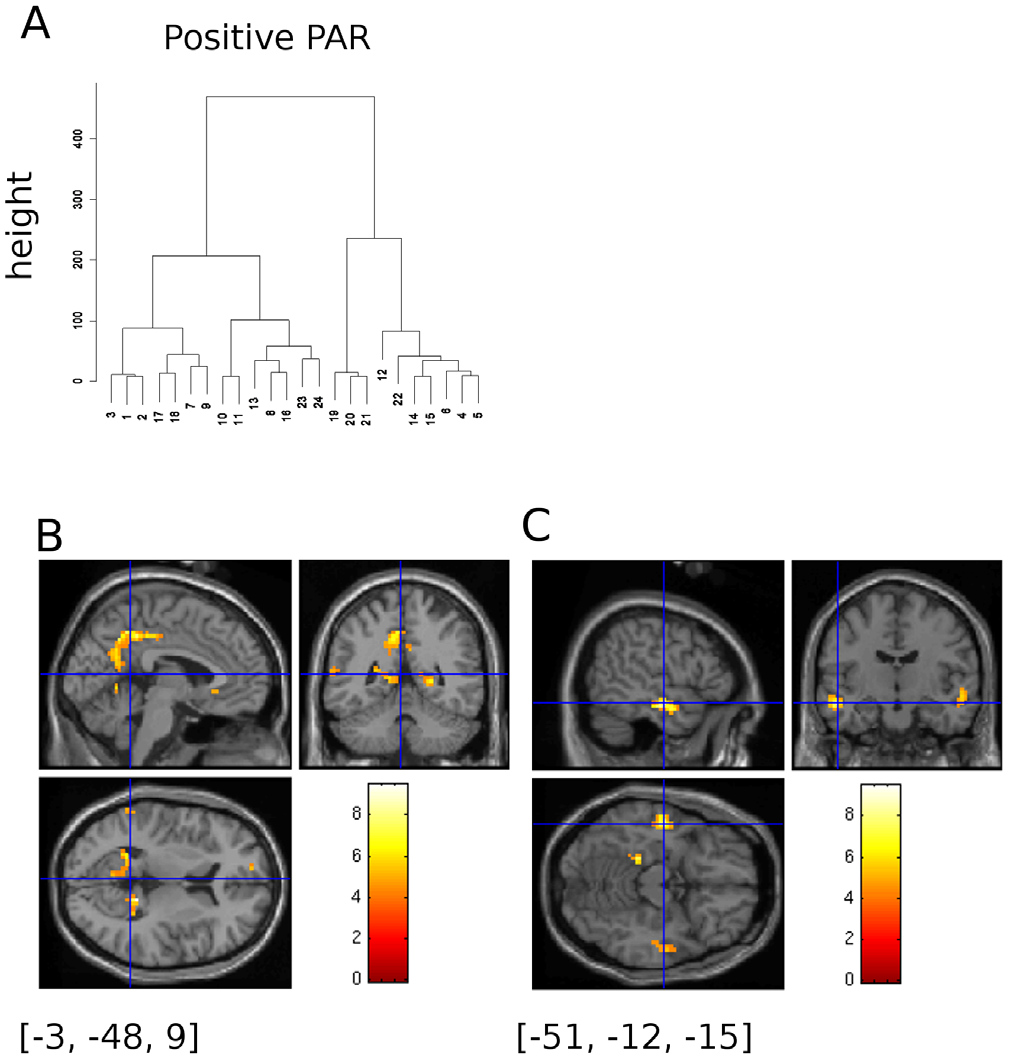
Positive Regression of BMI_PAR_ with BOLD. A)Cluster dendrogram for (X,Y,Z) peak BOLD activations sites for the positive regression of BMI_PAR_ with BOLD signal intensity. B)Bilateral cingulate gyrus activation. The cross hair was positioned between the two posterior cingulate gyrus activations at [−3, −48, 9] so both can be seen. Crosshair location in untransformed MNI co-ordinates. C)Bilateral anterior temporal lobe activation within the medial temporal gyrus (BA21). The cross hair was positioned at [−51, −12, −9] untransformed MNI coordinates, table 2 Cl5(Sx = 10).

The results of a positive regression of BODY and BMI_PAR_ values are shown in table 2. The analysis was as for the negative BOLD regression with BMI_PAR_ and again the k-means clustering tree of the corrected X,Y & Z co-ordinates (figure 4a) was cut to yield 9 clusters; these are shown in column Cl of table 2 , labelled 1–9. The preponderance of BOLD activity observed was in or near the posterior cingulate gyrus of the right hemisphere (figure 4B). The largest region activated (Table 2, Cl = 1,Sx = 1–3) had an extended volume of activation engulfing the parahippocampal gyrus and the posterior caudate. Cluster 4 (Sx = 8, 13, 16) seems to be a companion activation to this in the left hemisphere. Anterior cingulate activation was also evident, cluster 2 showing the close association between the left and right cortices; one location in this group was reported within the superior frontal gyrus (Cl = 2, Sx = 22, BA9). Visual association areas within the occipito- temporal route were activated too, Clusters 5, 7, 8 & 9 predominantly being reported as BA 18, 19, 20, 21, & 22; bilateral anterior temporal lobe activation within the medial temporal gyrus is shown in Figure 4C. A single relatively strong activation was seen in the superior frontal gyrus (cluster 6, Sx = 12, [−21.0, 13.5, 51.1] Talairach transformed coordinates; figure not shown), suggesting the engagement of the frontal eye fields as observed with the previous contrast.

**Table 2:** Positive regression of BODY and BMI_PAR_ (otherwise as in table 1).

Cl	Sx	K	cPu	FDR	T	EqZ	Punc	X	Y	Z	H	Structure	BA
1	1	81	0	0.019	9.47	4.84	0	12.65	−41.75	8.64	R	Posterior Cingulate	BA 29
1	2	(81)		0.02	6.73	4.15	0	18.27	−44.05	3.12	R	Parahippocampal Gyrus	BA 30
1	3	(81)		0.028	5.67	3.8	0	20.91	−42.32	14.13	R	Caudate	Caudate Tail
2	4	45	0	0.019	9.22	4.79	0	7.53	43.69	0.44	R	Anterior Cingulate	BA 32
2	5	(45)		0.026	5.94	3.9	0	13.02	35.02	2.41	R	Anterior Cingulate	*
2	6	(45)		0.033	5.24	3.64	0	4.75	27.2	−3.88	R	Anterior Cingulate	BA 24
2	14	11	0.015	0.021	6.61	4.12	0	−6.44	23.94	1.03	L	Caudate	Caudate Head
2	15	(11)		0.041	4.74	3.43	0	−11.94	29.82	−1.21	L	Anterior Cingulate	BA 24
2	22	13	0.009	0.029	5.52	3.74	0	−12.17	47.29	22.06	L	Superior Frontal Gyrus	BA 9
3	7	353	0	0.019	8.98	4.74	0	−7.17	−44.52	37.77	L	Cingulate Gyrus	BA 31
3	9	(353)		0.019	7.55	4.39	0	4.1	−59.78	20.3	R	Precuneus	BA 23
4	8	(353)		0.019	8.39	4.6	0	−20.72	−50.22	9.98	L	Posterior Cingulate	BA 30
4	13	18	0.003	0.019	7.31	4.33	0	−20.36	−33.9	−12.79	LCb	Culmen	*
4	16	7	0.044	0.024	6.08	3.95	0	−29.09	−39.52	16.26	L	Insula	BA 13
5	10	69	0	0.019	8.6	4.65	0	−48.07	−11.39	−11.13	L	Sub−Gyral	BA 21
5	11	(69)		0.02	7.06	4.25	0	−45.21	−5.3	−15.91	L	Middle Temporal Gyrus	BA 21
6	12	29	0	0.019	7.87	4.48	0	−21.01	13.46	51.14	L	Superior Frontal Gyrus	BA 6
7	17	40	0	0.027	5.81	3.85	0	3.99	−77.07	24.07	R	Cuneus	BA 18
7	18	(40)		0.043	4.66	3.39	0	−1.69	−86.21	31.21	L	Cuneus	BA 19
8	19	41	0	0.029	5.59	3.77	0	51.8	−12.44	−4.13	R	Superior Temporal Gyrus	BA 22
8	20	(41)		0.039	4.85	3.47	0	43.62	−8.55	−14.71	R	Sub-Gyral	BA 21
8	21	(41)		0.049	4.36	3.25	0.001	46.38	−16.95	−15.46	R	Sub-Gyral	BA 20
9	23	21	0.001	0.035	5.08	3.57	0	−59.57	−44.43	9.87	L	Superior Temporal Gyrus	BA 22
9	24	13	0.009	0.042	4.69	3.41	0	−43.26	−71.76	29.18	L	Angular Gyrus	BA 39

### Positive Regression of WHR with Body

No activations reached the predetermined criteria for detection (false discovery rate p(FRD)<0.05, cluster extent K>6, cPu<0.05), or when the FDR criterion was reduced to P<.1 ; no figure is provided. Based on uncorrected cluster statistics alone (cPu<0.05), three areas of activation were suggestive of underlying brain activity. The largest region was in the inferior parietal lobule of the left hemisphere ( (−37.86, −49, 47.45), BA 40, and (−40.65, −40.07, 51.14), BA 40) with a small corresponding region in the right hemisphere (39.79, −41.01, 57.82; BA 40).

### Negative Regression of WHR with Body

A negative regression of WHR with BOLD signal found no region that met the false discovery rate and cluster level statistical criteria for detection. One activation was observed that met the cluster level criterion (cluster extent >7, p<.05) in the right superior temporal gyrus (−42, 12.1, 57.0; BA22, cluster equivalent K  = 10, p = 0.02); no figure is provided. This is suggestive of high order visual processing of WHR-specific information within the temporal lobe, but the evidence is inconclusive.

### Contrast between Judge and Body

The level of BOLD response was compared in a simple main effects contrast between JUDGE and BODY. The body-shaped pictures were visible in both periods. The k-means clustering tree of the corrected X,Y & Z co-ordinates (figure 4a) was cut to yield 10 clusters. The results are dominated by visually activated regions shown in tables 3 and 4 as clusters Cl 1 & 3 (left hemisphere) Cl 2 (RH). In the frontal lobe there were some small regions within BA6 with significantly increased BOLD activity during the BODY interval compared to the JUDGE interval (Cluster 5); again we infer these were associated with activity in the frontal eye fields.

**Table 3:** Simple Effects contrast JUDGE –BODY.

Cl	Sx	K	cPu	FDR	T	EqZ	Punc	X	Y	Z	H	Structure	BA
1	1	506	0	0.002	11.23	5.17	0	−29.11	−89.05	3.46	L	Middle Occipital Gyrus	BA 18
1	2	(506)		0.002	8.67	4.67	0	−45.6	−76.73	−6.47	L	Middle Occipital Gyrus	BA 19
1	3	(506)		0.002	7.95	4.5	0	−37.28	−79.57	−6.59	L	Inferior Occipital Gyrus	BA 19
2	4	1676	0	0.002	10.6	5.06	0	29.16	−86.81	7.36	R	Middle Occipital Gyrus	BA 18
2	5	(1676)		0.002	10.26	5	0	31.92	−78.71	10.87	R	Middle Occipital Gyrus	BA 19
2	6	(1676)		0.002	9.5	4.85	0	43.2	−63.74	1.67	R	Inferior Temporal Gyrus	*
3	7	599	0	0.002	9.28	4.8	0	−40.71	−37.8	56.76	L	Inferior Parietal Lobule	BA 40
3	8	(599)		0.002	8.85	4.71	0	−51.64	−25.51	46.93	L	Postcentral Gyrus	BA 2
3	9	(599)		0.002	8.05	4.52	0	−37.91	−26.63	57.87	L	Precentral Gyrus	BA 4
3	24	82	0	0.003	7.04	4.25	0	−23.98	−64.78	43.68	L	Superior Parietal Lobule	BA 7
3	25	(82)		0.009	5.24	3.64	0	−18.56	−60.27	55.01	L	Precuneus	BA 7
4	10	332	0	0.002	8.06	4.52	0	−4.33	−5.67	44.21	L	Cingulate Gyrus	BA 24
4	11	(332)		0.003	7.17	4.29	0	1.42	17.7	35.71	R	Cingulate Gyrus	BA 32
4	12	(332)		0.003	6.62	4.12	0	1.29	5.74	42.68	R	Cingulate Gyrus	BA 24
5	13	78	0	0.002	7.8	4.46	0	28.73	−10.47	63.23	R	Precentral Gyrus	BA 6
5	14	(78)		0.031	3.79	2.97	0.001	31.68	−11.97	49.62	R	Precentral Gyrus	BA 6
5	21	100	0	0.002	7.56	4.39	0	−7.47	−13.86	70.4	L	Medial Frontal Gyrus	BA 6
5	22	(100)		0.005	6.06	3.94	0	0.9	−10.85	68.13	L	Medial Frontal Gyrus	BA 6
5	23	(100)		0.016	4.5	3.32	0	9.11	−17.26	75.76	NG		

Contrast between JUDGE and BODY (otherwise as in table 1).

**Table 4:** Simple Effects contrast JUDGE –BODY.

Cl	Sx	K	cPu	FDR	T	EqZ	Punc	X	Y	Z	H	Structure	BA
6	15	75	0	0.002	7.8	4.46	0	9.94	−2.87	14.98	R	Caudate	Caudate Body
6	16	(75)		0.009	5.17	3.61	0	9.92	−11.25	14.19	R	Thalamus	Ant. Nucleus
6	17	(75)		0.023	4.14	3.15	0.001	1.63	−8.15	11.64	R	Thalamus	*
6	28	23	0.016	0.003	6.65	4.13	0	−23.08	2.19	−6.72	L	Lentiform Nucleus	Putamen
6	33	49	0.001	0.009	5.15	3.6	0	−12.32	−8.61	16.77	L	Thalamus	*
6	34	(49)		0.011	4.95	3.52	0	−12.19	0.56	9.53	L	Lentiform Nucleus	Putamen
7	18	144	0	0.002	7.62	4.41	0	−39.85	12.41	4.78	L	Insula	BA 13
7	19	(144)		0.004	6.31	4.02	0	−48.17	4.33	1.17	L	Superior Temporal Gyrus	BA 22
7	20	(144)		0.019	4.32	3.23	0.001	−39.94	−7.42	5.6	L	Insula	BA 13
7	32	38	0.003	0.007	5.43	3.71	0	−45.71	−25.46	17.31	L	Insula	BA 41
8	26	111	0	0.003	6.98	4.23	0	37.92	12.27	3.38	R	Insula	*
8	27	(111)		0.007	5.52	3.75	0	46.23	6.63	2.99	R	Insula	BA 13
8	29	171	0	0.004	6.46	4.07	0	37.58	1.53	26.68	R	Precentral Gyrus	BA 6
8	30	(171)		0.008	5.28	3.65	0	46.05	2.53	16.11	R	Insula	BA 13
8	31	(171)		0.022	4.19	3.17	0.001	45.73	0.18	40.2	R	Middle Frontal Gyrus	BA 6
8	35	17	0.034	0.009	5.15	3.6	0	54.11	−21.44	30.19	R	Postcentral Gyrus	BA 2
9	36	7	0.154	0.016	4.5	3.32	0	−31.71	46.61	29.78	L	Superior Frontal Gyrus	BA 9
9	39	10	0.093	0.029	3.86	3.01	0.001	−40.18	34.69	36.61	L	Middle Frontal Gyrus	BA 9
10	37	22	0.018	0.019	4.31	3.23	0.001	40.33	31.48	37.67	R	Middle Frontal Gyrus	BA 9
10	38	19	0.026	0.024	4.05	3.1	0.001	34.91	43.47	30.61	R	Middle Frontal Gyrus	BA 9

Contrast between JUDGE and BODY (otherwise as in table 1).

**Figure 5:**
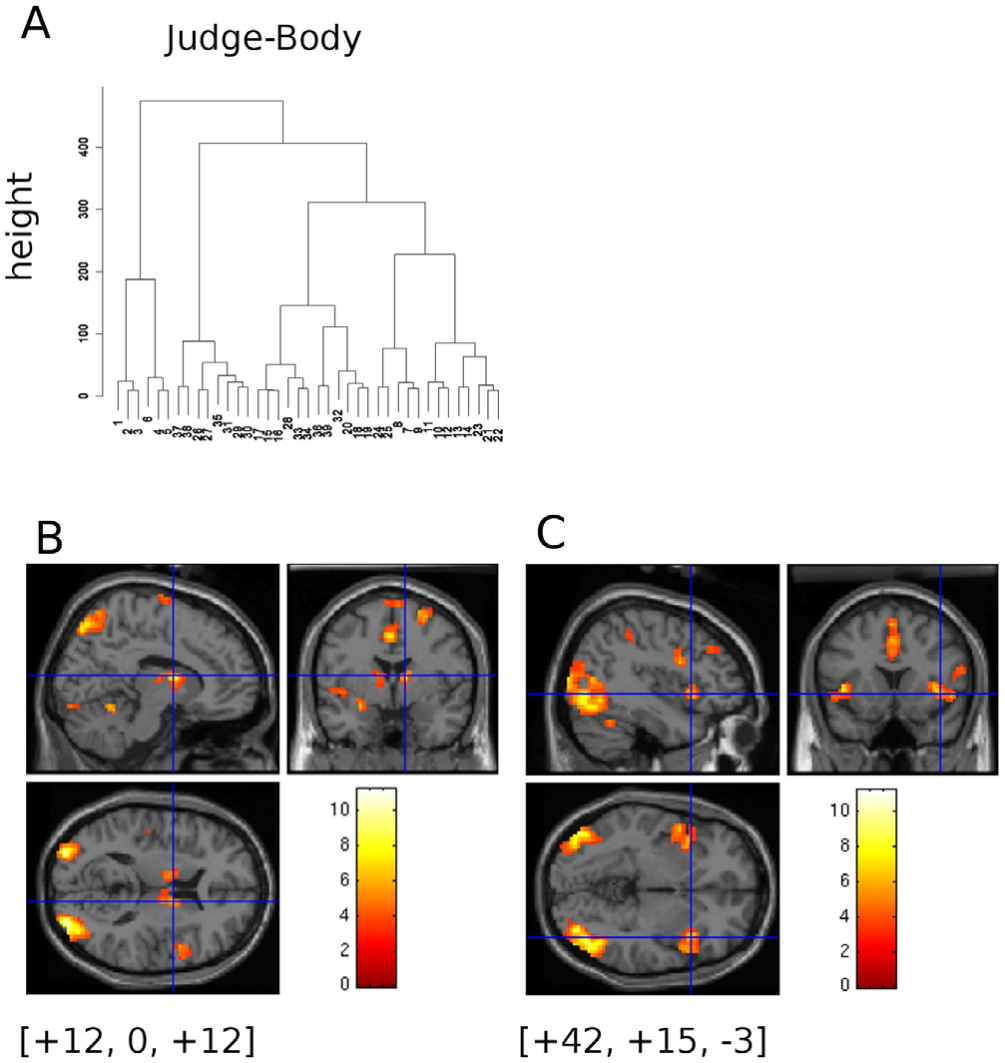
T-Contrast of BOLD signal between the intervals JUDGE and BODY. A)Cluster dendrogram for (X,Y,Z) peak BOLD activations sites for the contrast between the period of participant response (JUDGE) and participant body evaluation (BODY). B)Bilateral visual cortical activations in posterior parietal and lateral occipital regions. Bilateral activation in and around the caudate nucleus is present at the position of the cross hairs: [+12, 0,+12] untransformed MNI co-ordinates. C)At a lower plane, bilateral activation foci are present in the insula cortex. Cross hairs: [+42, +15, −3] untransformed MNI co-ordinates.

In addition to the general visual cortical activation, medial cortical and sub-cortical structures also showed significantly increased BOLD signal during the BODY interval. Clusters 4 & 6 are a group of BOLD activation loci identified by the clustering algorithm on the anterior midline. These clusters are dominated by cingulate cortex activation but they also include the underlying midbrain structures nearby: the caudate nucleus, the lentiform nucleus and the putamen, hinting that these brain regions form a network involved in this task. Figure 5 B shows the bilateral BOLD activation in the caudate nucleus; the cross-hairs are located at the right caudate activation ([9.9, −2.9, 15.0] Corrected coordinates, Cl 6, Sx = 15). Furthermore, bilateral activation of the insula cortex was detected (Cl = 7 & 8). These regions are the bilateral anterior lateral BOLD activations shown in figure 5 C axial and tangential sections.

### Negative Regression of Body on Participant Rating

When participant rating was modelled with a negative regression on the BOLD response BODY only one region met the statistical criteria, a cluster within the cingulate gyrus BA24 ( [−15.4, −2.8, 44.3] corrected coordinates, Sx = 1, no figure is provided) (see table 5). If the false discovery criterion is relaxed so that activations with p(FDR) <0.1 then several small regions of parietal lobe activations may be inferred within BA7, BA40, BA2. On the same basis minor activations of medial frontal gyrus (−7.2, −14.6, 48.7; BA6) and precentral gyrus (48.9, 3.3, 8.12; BA44) can be seen. These activations are perhaps related to visuo-motor activity. Of more interest, one small region (23 voxels extent, Sx = 7) of activation in the insula cortex was observed under the relaxed statistical criteria ([4.08, −45.8, −23.4], corrected coordinates, Sx  = 9). As the insula is known to be involved in emotional processing of negatively valenced stimuli then the reduced BOLD activation with increasing participant rating is weak evidence that this region is responding preferentially to negatively valenced stimuli. There were no regions that showed a positive regression of RATING on BODY.

**Table 5:** Negative regression of BODY and RATING.

Cl	Sx	K	cPu	p(FDR)	T	EqZ	X	Y	Z	H	Structure	BA
1	1	29	0	0.05	8.56	4.64	−15.43	−2.82	44.29	L	Cingulate Gyrus	BA 24
1	2	32	0	0.09	7.35	4.34	−7.2	−14.56	48.72	L	Medial Frontal Gyrus	BA 6
2	4	13	0.005	0.09	7.06	4.25	48.93	3.3	8.12	R	Precentral Gyrus	BA 44
2	5	34	0	0.09	7.03	4.24	53.96	−25.28	40.63	R	Postcentral Gyrus	BA 2
2	6			0.09	6.23	4	56.95	−20.93	24.88	R	Inferior Parietal Lobule	BA 40
3	7	23	0	0.09	6.5	4.08	−45.81	−23.45	25.6	L	Insula	BA 13
4	9	73	0	0.09	6.43	4.06	−21.31	−51.87	55.76	L	Sub-Gyral	BA 7
4	10			0.09	6.22	3.99	−24.2	−52.64	63.74	L	Superior Parietal Lobule	BA 7
5	12	14	0.004	0.09	6.26	4.01	9.23	−52.03	56.26	R	Precuneus	BA 7

Negative regression of BODY and RATING (otherwise as in table 1). No activations were obtained above threshold with the FDR p<.05 statistical criterion. The statistics shown are for an uncorrected voxel threshold P<.001 and cluster extent >7. Activations with uncorrected cluster probability cPu >.05 were removed. Missing elements in column Sx reflect activations that failed the cluster statistical criterion. There were no activations for the positive regression.

## Discussion

Our behavioural results suggest that BMI_PAR_ is closely correlated with attractiveness judgments and that WHR is not. This is consistent with previous behavioural and eye-movement studies which suggest that the primary predictor of attractiveness judgements for a female body is BMI, and that other physical features, such as WHR, have a much weaker effect on these judgements (e.g. [1], [10], [21]). Consistent with these behavioural results, our imaging results show that altering the apparent overall body mass of the bodies (as indexed by the BMI_PAR_) modulates activity in both the higher visual areas and in neural areas which form part of the brain reward system including the caudate nucleus. Caudate activation has been observed with a range of rewards including cocaine [31], nicotine [32], money [33], and feedback on performance on behavioural tasks [34]. By comparison, modulation of the WHR produced no significant change in BOLD activity.

In this study we used both male and female participants, whereas the Platek & Singh study used just male participants. However, previous studies have shown extremely high correlations (correlations greater than 0.95) between the ratings of male and female participants in attractiveness ratings of female bodies (e.g. [2], [35]–[38]). This is a degree of correlation no different than the correlation between two groups of the same gender (i.e. males versus males or females versus females), strongly suggesting that both genders assess female bodies in the same way. This is predicted by mate selection theory which postulates that individuals will not only be able to judge the attractiveness of members of the opposite sex, but also will know their own attractiveness relative to other members of the same sex (e.g., [39]). This information allows an individual to concentrate on potential partners of the same attractiveness as themselves, thus avoiding both unsuccessful courtship of a more attractive partner (potentially wasteful in time and resources) and accepting a less attractive partner (with a potentially negative impact on future reproductive success). Thus, both male and female observers should assess the bodies in the same way.

The fMRI results show that the appraisal of body shape involves a complex network of brain regions, with aspects of the task related to visual shape processing generally activating posterior regions; activation of the extra-striate body area (EBA) was seen, but not the fusiform body area (FBA). The EBA is believed to sensitive to body parts and the FBA to the whole body [40], forming part of a system which has been suggested to be analogous to the face recognition system [41]. As the stimuli used here did not include the whole body (see figure 1), but were centred on the torso, it is possible that they were not the optimal stimuli for the FBA.

Studies which have looked at judgements of attractiveness using either photographs of bodies (e.g.,[2], [35], [37], [42]–[45]), video clips of bodies (e.g.[20], [37]) or laser scanned 3D bodies (e.g. [11], [46]) have found that BMI is the primary predictor of attractiveness and health judgements by both male and female observers. Additionally in an eye-movement study, Cornelissen et al. [10] asked people to rate images for BMI, WHR and attractiveness. The areas of the body fixated when judging BMI were also fixated when estimating attractiveness, suggesting an assessment of BMI is part of the judgements made when rating attractiveness. The areas fixated when estimating WHR were not included in the areas fixated when estimating attractiveness, suggesting WHR is not directly assessed in attractiveness judgements. The importance of BMI is not only true of western populations, but seems to apply cross-culturally (e.g. [43], [47]–[49]). The importance of BMI in attractiveness judgments makes sense in an evolutionary context as it provides a reliable cue to female health (e.g. [50], [51]) and reproductive potential (e.g. [52]–[55]). However, this is not to say that WHR plays no role in attractiveness judgements. Several studies which have tried to separate out the relative importance of different physical features in predicting attractiveness judgements have found a weak role for WHR (e.g. [1], [37]). Additionally, several studies have explored which physical variables in their female participants predict their ratings of male faces and found significant correlations between WHR and their choices ([56]–[58]). This may imply that WHR may be linked to the female participant's estimate of their own attractiveness. Thus, it is quite possible that using images of female bodies with a much wider range of WHR, it would have been possible to find a correlation between this feature and our participants' attractiveness judgements and a corresponding activation of brain reward mechanisms. However, given the weaker predictive power of WHR for attractiveness judgements, it would be consistent to expect a correspondingly weaker activation of the brain's reward centres with modulating WHR even over a wide range of shapes.

Our behavioural results show a significant correlation between the BMI_par_ of the bodies in our images and their attractiveness ratings by our observers. Our results also show a significant correlation between BMI_par_ and activation of part of the brain reward areas in our observers brains, but they do not show a correlation between the ratings of attractiveness and BOLD activation of brain reward areas unless we relax our correction factors for multiple comparisons in the analysis (like [19]). This may be because the simple activation of the reward centres then has to be filtered through more complex cognitive decision making mechanisms to generate a rating response, which may weaken a simple 1 to 1 mapping of reward reaction to rating response so that it only meets a lower level of significance. It may also be that any variance in the ratings will act to reduce the detectability of the relationship between ratings and brain activation. Although images with a particular BMI_PAR_ value may always produce the same BOLD activation, the corresponding behavioural rating of the image over the course of these presentations will fluctuate around a mean value. If this fluctuation is relatively high, then it becomes harder to model the relationship between the brain's response and the ratings even though the BOLD response itself is reliably linked to the image.

Our experimental results show that increasing apparent body mass, indexed by BMI_PAR_, is strongly related to decreased BOLD response within the caudate nucleus bilaterally. Other mid-brain nuclei similarly modulated but to a lesser degree are the putamen and the anterior thalamic nucleus. The contrast between JUDGE and BODY showed BOLD activation was decreased in these midbrain structures, and within the insula cortex bilaterally (Figure 4B & C), during body shape appraisal (BODY) with respect to the later rating period (JUDGE). An increasing BMI has consistently been correlated with decreasing attractiveness ratings for female bodies (e.g. [1], [2]). The decrease in activation in caudate nucleus in response to decreasing preference is consistent with the results which have suggested reduced activity in the caudate nucleus with negative reward in a decision making task [59] and reduced activity in the caudate nucleus in response to aesthetically less pleasing representational and abstract paintings [5], [60]. Additionally, there is reduced activity in caudate nucleus in depressed patients relative to normal controls [61], [62]. One feature of depression is a decrease in the ability to experience pleasure and reward (anhedonia). A comorbidity between depression and Anorexia Nervosa (AN) has long been established, indeed part of the diagnostic criteria of AN is a disturbed body image [63]. The reduced activity in the caudate nucleus might be implicated in the impaired ability to accurately evaluate an attractive and healthy body (perceptions of attractiveness and health are very highly correlated) which has been demonstrated in the Anorexic observers [14], [15]. Activation within the lentiform nucleus and the anterior thalamus was also seen and this may point to this group of related sub-cortical nuclei being involved in processing body-shape judgements.

Our results suggest that BMI modulates reward mechanisms in the brain and we infer that this may have important implications for judgements of ideal body size in eating disordered individuals. Controlling body size through restricting diet, often augmented by excessive exercise and/or purging and vomiting, is a central feature of Anorexia Nervosa (e.g.[64], [65]). Sufferers are constantly checking their body size in the mirror and their weight on the scales. Behavioural studies have shown Anorexic observers prefer a significantly lower ideal body size for both their own, and other women's bodies [14], [15] and the progressive activation of the brain's reward mechanisms as BMI decreases shown in our study provides a potential mechanism by which this activity is rewarded and reinforced.

Further work will be needed to conclusively demonstrate that the caudate is important in judgements related to body size and shape, and whether there is a more extensive sub-cortical network of brain regions within which the caudate participates. This suggests that a further investigation to study individual variability with a focus on this sub-cortical network as region of interest would be a fruitful avenue for future research. Beyond this we look towards studies that seek to determine whether differences in activity in the nuclei around the caudate head play a role in the development of eating disorders.
